# Effectiveness of hospital-to-home transitional care interventions and consultation for implementation in Sudan: a scoping review of systematic reviews

**DOI:** 10.3389/frhs.2023.1288575

**Published:** 2023-12-14

**Authors:** Asma Mohamedsharif, Mohammed Elfeaki, Rayan Bushra, Armin Gemperli

**Affiliations:** ^1^ Faculty of Health Sciences and Medicine, University of Lucerne, Lucerne, Switzerland; ^2^ Directorate of Quality, Development and Accreditation, Federal Ministry of Health, Khartoum, Sudan; ^3^ Department of General Medicine, Ibrahim Malik Teaching Hospital, Khartoum, Sudan; ^4^ Center of Primary and Community Care, University of Lucerne, Lucerne, Switzerland; ^5^ Swiss Paraplegic Research, Nottwil, Switzerland

**Keywords:** chronic disease, integrated healthcare, primary care, transition of care, hospital discharge, low-income country, low-resource setting

## Abstract

**Background:**

Hospital discharge is often associated with a lack of continuity resulting in fragmented care, particularly in low-income countries. As there is limited information about interventions in these countries and no study evaluating the effectiveness of hospital discharge interventions, we conducted a scoping review to identify effective hospital-to-home transitional care interventions and explore their applicability in a low-income country (Sudan).

**Methods:**

Our scoping review of systematic reviews and meta-analyses classed interventions as effective, ineffective, undesirable, or uncertain, based on the quality of their evidence and their estimated effects on the following outcomes: readmission rates, mortality, costs, quality of life, and adverse outcomes) and certainty of evidence. Our authors from Sudan used the SUPPORT summary tool to determine if three effective interventions could be implemented in Sudan.

**Results:**

Out of 3,276 articles that were identified, and 72 articles were reviewed, 10 articles has been included in the review. Seven interventions were classified as effective, one as ineffective, and none with undesirable effects. Eight interventions were classified as having an uncertain effect. The effective interventions were composed of home visits, information and communication technology (ICT), case manager models, multidisciplinary teams, and self-management support.

**Conclusions:**

The finding of this study suggested that a combining two to four interventions can improve enhance hospital-to-home transitional care. Effective interventions are composed of home visits, ICT, case manager models, multidisciplinary teams, and self-management support. The implementation of these interventions in Sudan was found to be undermined by contextual factors such as inadequate human resources, telecommunication instability, and inequality in accessibility. These interventions could be tailored based on an in-depth understanding of the contextual factors in low-income countries that influence implementation.

**Systematic Review Registration:**

https://osf.io/9eqvr/, doi: 10.17605/OSF.IO/9EQVR

## 1. Introduction

Transitional care comprises actions that ensure the coordination and continuity of healthcare when patients transfer between locations or levels of care (1). Poor transitional care during, e.g., hospital discharge can harm patients and increase healthcare costs (2). When care continuity is disrupted at hospital discharge, patients have suboptimal outcomes including adverse events, hospital readmissions, or even death (2–4); potentially preventable 30-day hospital readmissions cost US Medicare about $12 billion annually (5, 6). Poor coordination and fragmentation of care are common in fragile health systems, where readmission and mortality rates after hospital discharge are especially high (7). There is a growing burden of chronic non-communicable diseases in low and middle-income countries (LMICs), which are associated with multiple hospital admissions (8, 9). Additionally, the report shows that Sudan has fewer than seven beds per 10,000 population, compared to 23 beds per 10,000 in LMICs (9). In low-income countries like Sudan, lack of continuity of care is the result of significant gaps in healthcare service. Interventions that prevent hospital readmissions and reduce unnecessary healthcare service utilization are urgently needed (10). Transitional care can be improved by effective targeted interventions, including interventions based on well-defined models of person-centered care, clear responsibilities, accountability for communication during transitions of care, sufficient patient engagement in care planning and communication, increased access to complete and up-to-date health and social information, more opportunities for medication reconciliation, and adequate discharge planning (11).

A “transitional care strategy” comprises one or more interventions initiated before hospital discharge to ensure the safe and effective transition of patients from setting to setting, e.g., from the hospital to home (12). Transitional care strategies including Care Transitions Intervention (CTI), Transitional Care Model (TCM), Project Reengineered Discharge (Project RED), and Project Better Outcomes for Older Adults Through Safe Transitions (BOOST) have been successfully implemented and evaluated in patient populations and healthcare settings across the US (2, 13–15). Several published interventions has improved care transitions e.g., standardizing documentation, defining care pathways, discharge planning, and medication reconciliation practices (10–13).

Literature on the transition from hospital to home is common (12, 14, 16–18), but assessments of published interventions are rare, especially in low-income countries. Since most evidence on transitional care was generated in and on interventions designed for high-income countries (HICs), its applicability to LMICs (19) is unknown. Since the success of healthcare transition strategies depends on contextual factors, including patient needs and organizational culture (15), and since patients from specific cultural backgrounds may face additional challenges during care transitions due to language and cultural barriers or low health literacy (20, 21), we need to systematically assess the challenges posed by implementing such interventions in LMICs. An assessment should consider an intervention's applicability, equity, costs, and the resources and infrastructure needed to monitor and evaluate the intervention (22) and provide practitioners, policy makers, and researchers with a list of interventions that could be implemented in LMICs.

We drew on existing evidence from studies of interventions designed to improve the transition from hospital-to-home. Specifically, we (1) identified hospital-to-home transitional care interventions that effectively reduced mortality and hospital readmissions and (2) assessed the feasibility of implementing the identified effective interventions in Sudan.

## 2. Methods

**Figure 1:**
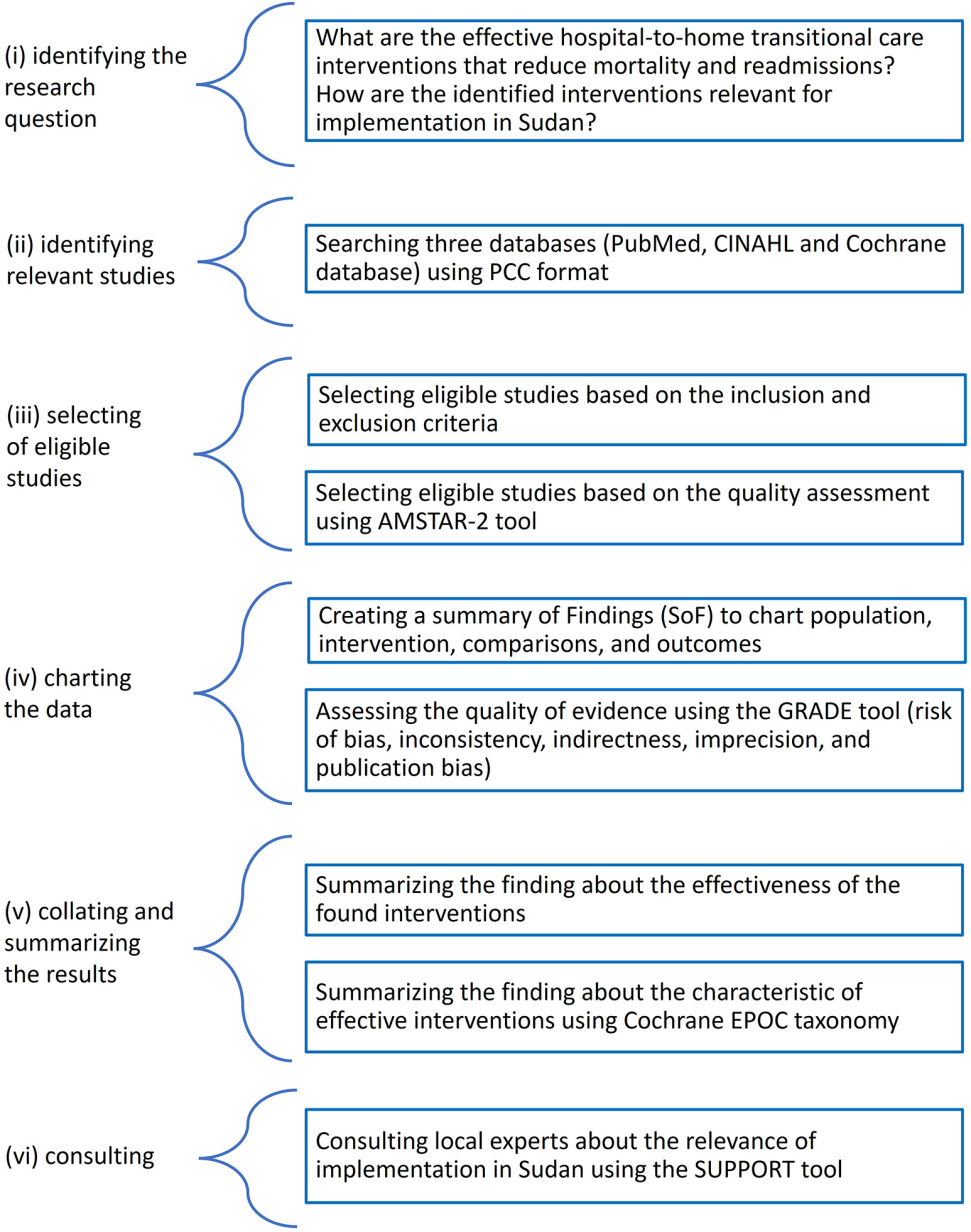
Summary of the scoping review methodology and the used tools.

We conducted a scoping review following the scoping review framework by Arksey and O'Malley: (i) identifying the research question, (ii) identifying relevant studies, (iii) selecting eligible studies, (iv) charting the data, and (v) collating and summarizing the results (vi) consulting (23). The reporting follows the PRISMA extension for Scoping Reviews (PRISMA-ScR) (24). The final protocol was registered prospectively with the Open Science Framework on 21 April 2021 (https://osf.io/7rbj6) (25) (Figure 1).

### 2.1. Search strategy

We searched three electronic online databases: Medline through PubMed; the Cumulative Index to Nursing and Allied Health Literature (CINAHL), and the Cochrane Database of Systematic Reviews. The search terms included variants (e.g., singular, plural, British/American spelling, truncation, etc.) and were selected according to the characteristics and functionalities of the specific database (Supplementary Data Sheet 1).

### 2.2. Eligibility criteria

We included English and Arabic language systematic reviews and meta-analyses of randomized controlled trials, nonrandomized controlled trials, controlled before-and-after studies, or interrupted time series studies published between 1 January 2000 and 15 March 2021. Inclusion criteria were studies in which adult or elderly patients were discharged from hospital to home. There were no restrictions on the reason for hospitalization or the location of the study. We excluded systematic reviews that included interventions focused on the transition of healthcare interventions between healthcare providers or healthcare settings.

Firstly, the titles and abstracts of the articles were screened to exclude those not relevant to the research question and to remove duplicates. In the second step, abstracts were screened to check for eligibility. For articles fulfilling the eligibility criteria in the title and abstract, the full articles were retrieved. Zotero and DistillerSR software were used (26, 27). To ensure accuracy whilst using the search strategy, two researchers (A.M. and A.G.) conducted the study selection process. A third, independent reviewer resolved disagreements between the two researchers. The results of the literature search were presented using the adapted PRISMA flow chart, which includes the number of citations screened, duplicates removed, full-text documents screened, and justification of excluding full articles.

### 2.3. Quality assessment of included studies

We used the AMSTAR-2 measurement tool to assess the quality assessment of all selected studies, as it is designed to appraise reviews that include both randomized controlled trials and non-randomized studies of interventions (28). AMSTAR-2 consists of 16 items: seven critical domains and nine non-critical domains. AMSTAR-2 does not generate an overall score but allows for an overall rating based on weaknesses in critical domains. We used AMSTAR-2 to rate reviews as high (no critical flaws with zero or one non-critical flaw), moderate (no critical flaws with more than one non-critical flaw), low (one critical flaw with or without non-critical flaws), or critically low (more than one critical flaw with or without non-critical flaws) quality. We included only moderate and high-quality studies to improve reliability. Two reviewers independently (A.M. and A.G.) assessed the quality of the studies. Disagreements were resolved by consensus.

### 2.4. Data extraction and analysis

We created 16 “Summary of Findings” (SoF) tables, between 1 and 3 for each review (29). The study of Takeda et al. (30) already included a SoF. The GRADEpro GDT software was used to generate the tables (31). Each SoF table includes population, intervention, comparisons, and outcomes that were identified as primary outcomes of the systematic review plus readmission rate, mortality, cost, quality of life, and adverse outcomes. We added a narrative description to the table if there were no meta-analyses. One reviewer (A.M.) assessed the quality of the body of evidence for outcome. We used the five GRADE considerations (risk of bias, inconsistency, indirectness, imprecision, and publication bias) (32). No further statistical synthesis (meta-analysis) was undertaken, due to the heterogeneity among reviews. The review findings were further classified into four categories:

1. **Effective**: interventions found to have desirable effects on at least one outcome with moderate- or high certainty evidence, and no undesirable effects with moderate- or high certainty evidence.
2. **Ineffective**: interventions found to have at least one outcome with little or no effect with moderate- or high certainty evidence, and no desirable or undesirable effects with moderate- or high certainty evidence.
3. **Undesirable**: interventions found to have at least one outcome with an undesirable effect with moderate- or high certainty evidence, and no desirable effects with moderate- or high certainty evidence.
4. **Uncertain**: interventions for which the certainty of the evidence was low or very low (or no studies were found) for all outcomes examined.

### 2.5. Qualitative synthesis

In this step, we identified the components of effective interventions. Two researchers were involved in qualitative synthesis (A.M. and A.G.). We used the Cochrane Effective Practice and Organization of Care (EPOC) taxonomy to provide a qualitative description of the components of effective interventions. EPOC categorized the components of the interventions according to changes in how, when, where, and by whom healthcare is organized and delivered (33). The taxonomy classifies the components of the interventions into five categories (and related subcategories) based on changes to the following: (1) how and when care is delivered; (2) where care is provided and changes to the healthcare environment; (3) who provides care and how the healthcare workforce is managed; (4) coordination of care and management of care processes; and 5) information and communication technology (34).

### 2.6. Consulting

The components of effective interventions were evaluated to determine their suitability for implementation in Sudan. We used a tool developed by the Supporting Policy-Relevant Reviews and Trials (SUPPORT) Collaboration (22, 35). This tool is a qualitative relevance assessment tool for the interventions within the reviews. The tool provides judgments about potential differences between where the research was conducted and its application in LMICs. The SUPPORT tool enables an assessment of interventions with respect to their applicability, impact on equity, economic considerations, and the necessity for monitoring and evaluation (36). The consultation was carried out by two researchers (ME, RB). Both researchers have a good understanding of the realities and constraints within health systems in Sudan. One person has macro-level policy and management insight as a director, the other is an internal medicine specialist with micro-level experience in service delivery. Each researcher made an initial, independent assessment, following the specific questions for each aspect of the SUPPORT tool (36), and then the findings were compiled and discussed.

## 3. Results

**Figure 2:**
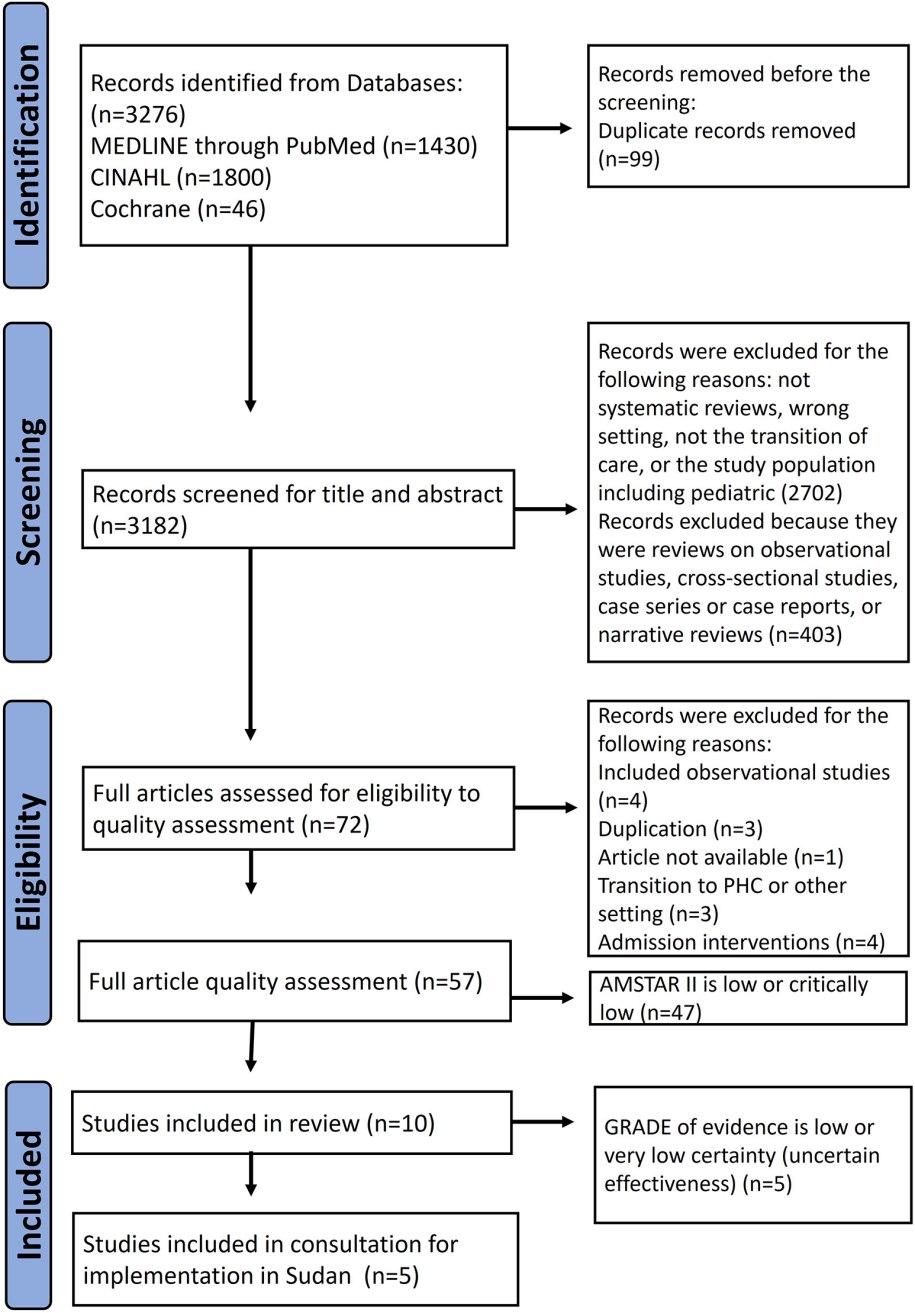
Study flowchart.

The literature search yielded 3,276 results, of which 99 were duplicates. Of these, we excluded 2,702 articles after evaluating the title and abstract for the following reasons: systematic review, wrong setting, non-transitional care intervention, or pediatric study population. We considered 72 articles as potentially eligible and screened them for methodological quality using AMSTAR-2 (Supplementary Data Sheet 2). We included the one high quality, and the nine moderate-quality reviews in our study and excluded 47 others (10 low and 37 critically low) as shown in Figure 2.

### 3.1. Characteristics of the included reviews

The reviews included in this study consisted of six systematic reviews with meta-analysis, two systematic reviews without meta-analysis, and two Cochrane reviews. The articles originated from Germany (*n* = 2), Canada, Belgium, the Netherlands, the UK, Italy, Japan, and China (*n* = 1, each). The included studies covered a publication period of 15 years (2006—March 2021). A total of 279 individual studies were included across all systematic reviews, including 251 randomized controlled trials (RCTs), 21 controlled clinical trials (CCT), and seven non-randomized control trials. Of the 279 trials, 250 (90%) were conducted in high-income countries, 29 (10%) in middle-income countries, and none in a low-income country (37). Four reviews focused on heart failure (30, 38–40), and two reviews focused on common chronic diseases (e.g., chronic obstructive pulmonary disease, chronic heart disease, chronic lung disease, diabetes, chronic kidney failure, or multi-chronic diseases) (18, 41), and one review focused on depression (42). Two reviews focused on geriatric patients (41, 43). Three studies investigated specific models of interventions to improve transition, such as pharmacist-led discharge counseling (44), virtual wards (VW) (40), and telephone follow-up (TFU) (45). All interventions were compared to usual care, which was not always explicitly described in the studies. Various outcome measures were used, such as variables related to clinical outcomes [e.g., hospital readmission within 30, 90 days, or 1 year; mortality; emergency department (ED) visits], patient-reported outcome measures (PROMs) (e.g., adverse effect; compliance; depression symptoms post-discharge; quality of life; patient satisfaction) and cost-effectiveness (e.g., the average cost per patient treated) (Table 1). Five reviews rated the studies according to a score for intervention intensity (18, 39, 41–43), which considers the length of intervention, number of contacts with the patient (42), or number of intervention components (39, 41–43). These components were either in the pre-discharge and post-discharge phases, or both (18, 43).

**Table 1:** Reviews characteristics.

Authors, year	Country of the included reviews	Target health issue	Number of included trials	Study design of the included trials	Countries of the included trials	Population	Intervention	Comparison	Primary outcome
Braet et al. (18)	Belgium	Not specific	51	51 RCT	USA (*n* = 24) Canada (*n* = 5), Brazil (*n* = 1), the Netherlands (*n* = 1), Israel (*n* = 1), Italy (*n* = 1), Spain (*n* = 2), Sweden (*n* = 1), United Kingdom (*n* = 3), Switzerland (*n* = 1), Austria (*n* = 1), New Zealand (*n* = 2), Australia (*n* = 2), China (*n* = 3), France (*n* = 1), Ireland (*n* = 1) and Croatia (*n* = 1)	18 years or older	Interventions had to be designed to ease the care transition from hospital to home or to prevent problems after hospital discharge	Usual care	Readmission within three months after discharge, patient satisfaction, return to emergency departments, and mortality
Takeda et al. (30)	UK	Heart disease	47	RCTs	USA (*n* = 11), Spain (*n* = 5), Turkey (*n* = 1), Australia (*n* = 1), New Zealand (*n* = 1), China (*n* = 3), UK (*n* = 4), Brazil (*n* = 1), Canada (*n* = 2), Switzerland (*n* = 1), Sweden (*n* = 4), Japan (*n* = 1), the Netherland (*n* = 2), Taiwan (*n* = 1), Austria (*n* = 3), Poland (*n* = 1) Iran (*n* = 2) and Italy (*n* = 3).	Adults aged 18 years and over, who had been admitted at least once to secondary care with a diagnosis of heart failure	Clinical service disease management interventions (defined as inpatient, outpatient, or community-based interventions or packages of care), directed specifically at people with heart failure	Usual care	Mortality due to heart failure, all-cause mortality, readmissions due to heart failure, all-cause readmissions, adverse effects, health-related quality of life, and costs or cost-effectiveness
Li Y et al. (38)	China	Heart failure	38	38 RCTs	USA (*n* = 16), Spain (*n* = 3), Germany (*n* = 1), Australia (*n* = 2), China (*n* = 3), UK (*n *= 2), Brazil (*n* = 2), Belgium (*n* = 1), Canada (*n* = 3), Sweden (*n* = 2), Ireland (*n* = 1), Iran (*n* = 1) and Italy (*n* = 1).	Patients older than 55 years during or within 1 week of an index hospitalization [i.e., initial hospitalization with a primary diagnosis of heart failure (HF) without preceding hospitalization for HF in 1 year] for HF	Transitional care interventions aim to prepare patients for the hospital-to-home transition with usual care and initiated the interventions within 2 weeks post-hospital discharge	Usual care	All-cause readmissions, HF-specific readmissions, ED visits, and hospital length of stay of subsequent readmissions within 6 months from the index hospitalization
Vedel and Khanassov (39)	Canada	Congestive heart failure	41	41 RCTs	USA (*n* = 19) Canada (*n* = 3), Brazil (*n* = 1), the Netherlands (*n* = 3), Italy (*n* = 3), Spain (*n* = 2), Sweden (*n* = 2), UK (*n* = 2), Switzerland (*n* = 1), Austria (*n* = 1), New Zealand (*n* = 1), Australia (*n* = 1) and China (*n* = 2)	Patients with congestive heart failure	Transitional care interventions (TCIs) on acute health service	Usual care	All-cause ED Visits, all-cause hospital readmission
Uminski et al. (40)	Japan	Heart failure and non-heart failure	10	10 RCTs	Italy (*n* = 2), Switzerland (*n* = 1), Canada (*n* = 2), Germany (*n* = 1), Belgium (*n* = 1), USA (*n* = 1) and Denmark (*n* = 2)	Populations with congestive heart failure, chronic kidney disease, chronic obstructive pulmonary disease, and high-risk medical conditions.	Post-discharge virtual wards	Usual care	Mortality and readmissions
Facchinetti et al. (41)	Italy	Older people with chronic diseases	30	30 RCTs	USA (*n* = 10) Canada (*n* = 1), Israel (*n* = 1), Italy (*n* = 1), Spain (*n* = 1), Sweden (*n* = 1), UK (*n* = 3), Switzerland (*n* = 1), New Zealand (*n* = 1), Australia (*n* = 3), China (*n* = 5), Japan (*n* = 1), and Croatia (*n* = 1)	Older patients (65 years) diagnosed with one or more chronic diseases, who were discharged home from the hospital	Continuity of care interventions provided by any healthcare professional during and after hospital discharge	Usual care	All-cause hospital readmissions
Morkisch et al. (43)	Germany	Geriatric patients	3	3 RCTs	Australia (*n* = 1), Spain (*n* = 1), and the USA (*n* = 1).	Geriatric patients	Transitional care model	Usual care	Readmissions
Bonetti et al. (44)	Brazil	Pharmacist-led discharge counseling	21 18 included in a meta-analysis	21 RCTs	USA (*n* = 6), Brazil (*n* = 1), the Netherlands (*n* = 1), Spain (*n* = 1), Canada (*n* = 1), Brazil (*n* = 2), Oman (*n* = 1), UK (*n* = 5), China (*n* = 1), Denmark (*n* = 2).	Patients of any clinical condition, gender, or age were included	Pharmacist-led discharge medication counseling vs. usual care	Usual care was defined as patients who received the usual treatment in regular practice	Readmissions and ED visits
Mistiaen and Poot (45)	Netherlands	Not specific	33	RCTs, controlled trials	Australia (*n* = 1), Canada (*n* = 7), the Netherlands (*n* = 1), Saudi Arabia (*n* = 1), the UK (*n* = 2), USA (*n* = 21).	All patients discharged from an acute hospital setting (including emergency departments and one-day-stay procedures) to home	Telephone follow-up (TFU) initiated by a hospital-based health professional (medical, nursing, social work, pharmaceutical,…) to a patient who is discharged to his/her home (including a relative's home)	Usual care, or other types of hospital follow-up	Assessment of the psychosocial health, physical health, adherence of patients to recommended care, and patient knowledge regarding
Holzinger et al. (42)	Germany	Depression	13	7 RCT and 6 Non RCT	UK (*n* = 1), USA (*n* = 3), Germany (*n* = 5), Switzerland (*n* = 1), Belgium (*n* = 1), Denmark (*n* = 1), and Italy (*n* = 1).	Patients with depression after psychiatric hospitalization	Discharge management strategies and post-discharge care interventions	Usual care	Readmissions and symptoms of depression

RCTs, randomized control trials; USA, United State of America; UK, United Kingdom; TCIs, transitional care interventions; ED, emergency department; HF, heart failure; TFU, telephone follow-up.

### 3.2. Effectiveness of the interventions

We assorted the interventions in the 10 reviews into 16 interventions, based on how the original reviews pooled the interventions for the meta-analysis or the descriptive analysis. We used the same name as stated in the original reviews to describe the intervention. For each intervention, we created SoF tables to assess its effectiveness. We found seven effective interventions and one ineffective intervention. We were uncertain about the effectiveness of eight interventions and identified no interventions with undesirable effects (Table 2 and Supplementary Data Sheet 3).

**Table 2:** Describe the interventions and their effectiveness classification.

Authors, year	Description of the interventions	Effectiveness classification based on GRADE assessment
Braet et al. (18)	Discharge planning interventions for adult patients	Uncertain about its effect
Takeda et al. (30)	Clinic-based interventions for heart failure patients	Ineffective intervention
	Multidisciplinary disease management program for heart failure patients	Effective intervention
	Case management program for heart failure patients	Effective intervention
Li et al. (38)	The interventions include clinic-based interventions, home visits, self-management, case management, and telemonitoring for patients with heart failure	Effective intervention
Vedel and Khanassov (39)	Discharge planning, self-management, home visits, and queuing strategies for patients with congestive heart failure	Uncertain about its effect
Uminski et al. (40)	Virtual wards (VW) for patients with heart failure which include home visits and telemonitoring	Effective intervention
	Virtual wards (VW) for effects for undifferentiated high-risk chronic disease patients which include home visits and telemonitoring	Uncertain about its effect
Facchinetti et al. (41)	Telemedicine, self-management, and case management for elderly patients	Effective intervention
Morkisch et al. (43)	Highly intensive, multicomponent, and multidisciplinary interventions for geriatric patients include comprehensive assessment, shared care, role expansion or task shifting, self-management, role expansion or task shifting, home visits, and queuing strategies	Effective intervention
	Moderately intensive, multicomponent, and multidisciplinary interventions for geriatric patients include comprehensive assessment, shared care, role expansion or task shifting, self-management, role expansion or task shifting, home visits, and queuing strategies	Effective intervention
Bonetti et al. (44)	Pharmacist-led discharge	Uncertain about its effect
Mistiaen and Poot (45)	Telephone follow-up (TFU) is the only intervention for discharged surgical patient	Uncertain about its effect
	Telephone follow-up (TFU) is the only intervention for discharged cardiac patients	Uncertain about its effect
	Telephone follow-up (TFU) is the only intervention for discharged cardiac surgery patients	Uncertain about its effect
Holzinger et al. (42)	Discharge management strategies and post-discharge care interventions for depressed patients	Uncertain about its effect

#### 3.2.1. Effective interventions

Virtual wards were considered effective interventions. They improved one-month hospital readmissions in patients with heart failure (Risk Ratio (RR) 0.60, 95% Confidence Interval (CI) 0.49–0.76) (40), and had an uncertain effect on all-cause readmissions, due to very low-certainty evidence (RR 0.86, 95% CI 0.67–1.11). It reduced all-cause mortality in patients with heart failure (RR 0.59, 95% CI 0.44–0.78), with an uncertain effect in an undifferentiated population (RR 0.98, 95% CI 0.84–1.15) due to very-low certainty evidence (40).

Case management for heart failure patients was classified as an effective intervention. It reduced hospital readmissions due to heart failure over 12 months by 36% compared to usual care (RR 0.64, 95% CI 0.53–0.78) and slightly reduced all-cause hospital readmissions over 12 months (RR 0.92, 95% CI 0.83–1.01) (30). Case management reduced all-cause mortality (RR 0.78, 95% CI 0.68–0.90), but showed an uncertain effect on mortality due to heart failure (RR 0.46, 95% CI 0.23–0.95). There was an uncertain effect of case management on quality of life and low-certainty evidence on cost-effectiveness (30).

The multidisciplinary disease management program for heart failure patients was classified as an effective intervention, as it reduced the risk of hospital readmission due to heart failure (RR 0.68, 95% CI 0.50–0.92) and all-cause over 12 months (RR 0.85, 95% CI 0.71–1.01) (30). It reduced all-cause mortality (RR 0.67, 95% CI 0.54–0.83), with an uncertain effect on mortality due to heart failure (RR 0.46, 95% CI 0.23–0.95). There was an uncertain effect of multidisciplinary disease management programs on quality of life. The effects of multidisciplinary disease management programs on costs or cost-effectiveness were reported with a low-GRADE rating. A study on the effects of multidisciplinary disease management programs on adverse events showed moderate quality in GRADE assessment, suggesting little or no difference in adverse effects between these multidisciplinary programs and usual care (30).

We categorized both the high and moderate-intensity multicomponent and multidisciplinary interventions for geriatric patients as effective interventions (43). A high-intensity intervention reduced hospital readmissions in three months from 42% to 29% in comparison to usual care. It did not reduce mortality, but improved quality of life, and reduced the cost of care by 460 US$ per patient. The moderate-intensity intervention showed a reduction in hospital readmission in the short term of 2 months (percent differences −54.4, *p* ≤ 0.05), 6 months (percent differences −42.4, *p* ≤ 0.05), and no difference at 12 months. It reduced mortality after 12 months from 30% to 13% (*p* = 0.017), showed no difference in the quality of life, but reduced costs (578 US$ per patient) (43).

We classified continuity of care interventions for the elderly, with a focus on the connection and coordination between providers, as effective interventions. They reduced short-term readmissions at one month (RR 0.74, 95% CI 0.65–0.84) and three months (RR 0.84, 95% CI 0.71–0.99) (41). Nevertheless, we were uncertain about their effect on long-term readmission at 3–6 months (RR 0.91, 95% CI 0.78–1.06) and at 6–12 months (RR 0.84, 95% CI 0.74–0.95) due to very low -certainty evidence (41).

We classified transitional care interventions for patients with heart failure as effective interventions. It reduced hospital readmissions due to heart failure (RR 0.89, 95% CI 0.82–0.97) and all-cause readmission rates (RR 0.78, 95% CI 0.68–0.89), without effect on the ED visits (RR 0.94, 95% CI 0.83–1.07) (38). It improved all-causes hospital readmissions (RR 0.92, 95% CI 0.87–0.98) and reduced ED visits (RR 0.71, 95% CI 0.52–0.98) in patients with congestive heart failure (38).

#### 3.2.2. Uncertain about the effectiveness

Discharge planning interventions for patients with congestive heart failure were found to have uncertain effects (39). They slightly reduce all-cause hospital readmissions (RR 0.92, 95% CI 0.87–0.98), with an uncertain effect on ED visits (RR 0.71, 95% CI 0.52–0.98), with low and very low quality of evidence, respectively (39). We classified discharge management strategies and post-discharge care interventions for depression as uncertain interventions. We were uncertain about its effect on reducing hospital readmissions (RR 0.65, 95% CI 0.42–1.01), reducing suicide rates (RR0.75, 95% CI 0.55–1.01), or improving quality of life, due to very low-certainty evidence (42). We were uncertain about their effect on hospital readmissions three months after discharge (RR 0.77, 95% CI 0.77–0.84), ED visits (18), and mortality (RR 0.75, 95% CI, 0.55–1.01). The pharmacist-led discharge counseling intervention had an uncertain effect on hospital readmissions despite statistically significant differences between the intervention and usual care groups (RR 0.86, 95% CI 0.76–1.00) and it also had an uncertain effect on ED visits (RR 0.70, 95% CI 0.54–0.91) (44). Telephone follow-up (TFU) as a single intervention had very low-certainty evidence. We are uncertain about its effectiveness on readmission in cardiac (RR 0.75, 95% CI 0.41–1.36) and surgical patients (RR 0.65, 95% CI 0.28–1.55) (45). There was also very low-certainty evidence about the effect of telephone follow-up on levels of anxiety, satisfaction, and compliance (45).

#### 3.2.3. Ineffective interventions

We found no probable difference in effect between clinic-based interventions for heart failure patients and usual care over 18 months in readmissions of patients with heart failure (RR 1.01, 95% CI 0.87–1.18) (30). Low-certainty evidence suggested that clinic-based interventions may result in little or no difference in all-cause readmissions (RR 0.87, 95% CI 0.68–1.10) and all-cause mortality (RR 0.87, 95% CI 0.68–1.10) (30). No difference to usual care in the quality of life was found in clinic-based interventions. Studies indicated that clinic-based interventions may reduce costs slightly, but the GRADE quality assessment was low for those studies (30).

#### 3.2.4. Undesirable interventions

There were no interventions that fall in the category of undesirable interventions.

### 3.3. Characteristics of effective interventions

**Figure 3:**
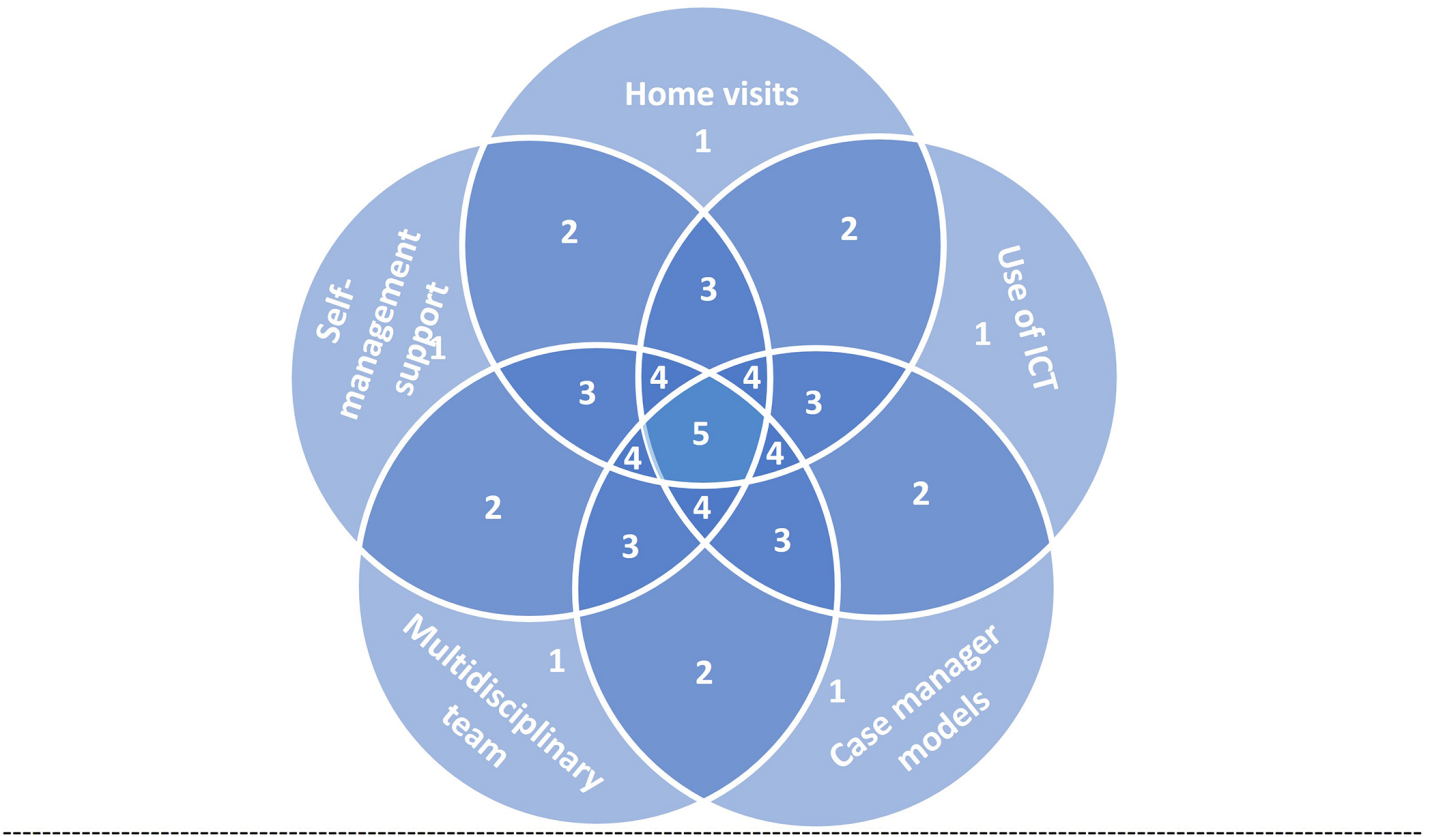
Conceptual diagram represents the possible combination of intervention components for effective interventions for the transition of care from hospital to home. Each field represent sets of interventions, 1, single component intervention; 2, intervention of two components; 3, intervention of three components; 4, intervention of four components; 5, intervention of five components. ICT, information and communication technology.

We categorized the components of effective interventions into five main categories based on EPOC: home visits, use of information and communication technology (ICT), case manager models, multidisciplinary team, and self-management support. These interventions were often combined in groups of two, three, or four to achieve optimal outcomes (Figure 3).

Home visits were a partial element of all effective interventions (30, 38, 40, 41, 43). They aim to provide participants with self-management advice (30), early communication with primary care providers and follow-up after discharge (43). Home visits were usually scheduled within the first week post-discharge with additional visits as required without prior scheduling. The number of scheduled home visits ranged between two to six in the first two months (41, 43). Home visits were usually combined with telephone calls (40, 41, 43).

Home visits and ICT were used by healthcare professionals to keep a relationship with patients and their caregivers and promote continuity of care (41, 43). All interventions used technology-based methods for communication, such as telephone hotline or telephone follow-up (30, 41, 43), structured telephone support (38), or the integration of telemedicine and case management components (38). Telephone follow-ups were mostly applied in the short term (1–3 months) (41). One review focused on the virtual wards (VW) (40), which provide patients with a period of intensive multidisciplinary team management at home, employing systems, staffing, and daily routines of a hospital ward, in addition, to incorporating telehealth and case management components.

Three reviews identified alternative role expansion and task shifting, such as a case manager model, consisting of active management and intense monitoring of high-risk patients with complex needs after discharge from the hospital in an integrated care system (30, 40). Case management is usually carried out by a nurse practitioner and typically involves home visits or telephone calls or both (30). Case managers play a vital role in patients' or family caregivers' training and coaching intending to enhance their self-confidence in monitoring and managing symptoms (41).

The multidisciplinary team is a holistic approach to the individual's medical, psychosocial, behavioral, and financial circumstances and typically involves several different professions working in collaboration (30). It can be an interdisciplinary hospital ward team but delivered in the patient's home (38). One review highlighted the follow-up of the patients by the same medical care team, to avoid interruption of the patient's plan of care and promote consensus on the patient's care plan between the patient and members of the healthcare (43).

Most interventions are aimed at promoting patient self-management (30, 38, 41, 43). Self-management support involves the implementation of educational and behavioral strategies to meet the patient's and family caregiver's learning needs to be related to an adequate and immediate response to the worsening of symptoms (43). Three reviews were on interventions to foster self-management support through psychotherapeutic interventions based on patient empowerment (41), implementations of educational and behavioral strategies (43), and educational intervention and individual peer support (38). Participants were sometimes given diaries or notebooks to aid self-management (30, 41, 43).

## 4. Discussion

We found that home visits, use of information and communication technology (ICT), case manager models, multidisciplinary teams, and self-management support were the most frequently described components of effective interventions to ease the transition from hospital to home. Effective interventions were bundled in groups of two, three, or four. A single intervention, for example, TFU (45) did not have a better effect on patient outcomes than a comprehensive intervention that combined TFU with home visits (called a virtual ward) (40). A recently published review of integrated care interventions concluded that combining such interventions is effective, particularly in reducing hospitalizations (46). The evidence for the effectiveness of interventions that focus on health providers' role expansion and task shifting was uncertain. One intervention introduced a transition coach who facilitates the transition between inpatient and outpatient settings (18). The other intervention was pharmacist-led discharge medication counseling (44). Role expansion and task shifting of healthcare providers may be beneficial, if the intervention is grounded in expanding their role to include case management and care coordination tasks (30, 40, 41).

### 4.1. Relevance to Sudan

The qualitative consultation on the implementation of effective interventions in Sudan has shown that not all of these interventions are currently integrated into the practice of transitioning care after hospital discharge within the existing Sudanese healthcare system. Self-management support and the multidisciplinary team are familiar with the Sudanese healthcare system, however, may still require further adaptation and improvement. Home visits, the use of ICT, and the case manager model are not standard services provided to patients after discharge from the hospital. Home visiting is used, for instance, as part of the reproductive health program and in an initiative to support people living with human immunodeficiency virus (HIV) (47, 48). Due to a variety of factors, including limited resources, inadequate infrastructure, a lack of trained healthcare professionals, and concern about inequality in accessing health services, these interventions may not be readily available or accessible to individuals in Sudan, and their implementation may require additional planning and support from the Federal Ministry of Health (FMOH) in Sudan.

#### 4.1.1. Applicability

The applicability of the described technologies of information communication interventions to Sudan is hampered by poor infrastructure in general and technological infrastructure in particular, which makes it difficult to monitor and exchange electronic information. This is potentially feasible in larger cities with access to telecommunications services. However, in rural areas, this is often not the case. Benefits are often limited to wealthier families and higher-income communities. Despite these limitations, there is ongoing communication between healthcare providers and patients using low tech like SMS messaging and WhatsApp in both urban and rural areas. Multidisciplinary teams, home visits and case manager models in low-income countries are hampered by shortages of human resources, particularly nurses. In Sudan, for example, there are 28.8 nurses and 21.4 doctors per 100,000 population (49), which is considered far below the threshold of 445 health workers per 100,000 population needed to deliver essential health services (50). These interventions require additional staff resources whilst the healthcare system struggles with the high turnover of qualified staff and irregular capacity-building activities. The educational level of the patients and their families were not examined in the studies, which is an important consideration, particularly for self-management support in low-income countries.

#### 4.1.2. Impact on equity

Applying home visits, ICT or case managers' intervention to LMICs will also raise the issue of inequity. The socioeconomic context of patients and their families could make the implementation of these interventions difficult. Out-of-pocket expenditure accounts for 67% of current healthcare expenditure in Sudan (51) and might only be affordable to wealthy patients.

#### 4.1.3. Economic considerations

Although some studies showed that these interventions reduced the cost, no conclusions can be drawn from the reviews on the costs or cost-effectiveness in LMICs. All interventions required additional resources such as rewards for health resources, training, supervisory staff time, and other associated costs. It is therefore important that economic evaluations be done as part of future studies.

#### 4.1.4. Need for monitoring and evaluation

All interventions need to be monitored and evaluated at the level of infrastructure, process and outcomes. Monitoring and evaluation of these interventions could be added as routine activities with existing staff. The main concern is that these interventions will face constraints when performed in a low-income country, e.g., technical, behavioral, and organizational/environmental challenges (52, 53).

The practical implication of this review is to optimize the use of low-tech ICT solutions, such as SMS messaging and WhatsApp, and design interventions that tailor them to Sudan contexts. This would include identifying strategies for implementing and scaling up successful interventions and integrating them into existing healthcare systems.

To address the challenges of implementing these components in Sudan, we suggest policies that include increased investment in health infrastructure, promotion of innovation and technology, and policies that promote universal health coverage such as national health insurance. Future studies should explore the contextual factors that influence the implementation process. Implementation studies are essential to validate the design of interventions and tailor them to improve transitional care in Sudan, considering the challenges of the local health system. Research could also explore cost-effective innovative ICTs that are used in other health areas, such as HIV, and adapt them to the area of transitional care from hospital to home.

### 4.2. Limitations and strengths

This review has several strengths and limitations. Our review only searched three databases, which may have resulted in some studies being missed. We also excluded studies that were not published in English or Arabic. We were uncertain about the quality of the evidence in half of the review studies. This uncertainty was mainly due to specific methodological limitations of the primary clinical trials. Due to the nature of the interventions, it wasn't possible to blind the participants and most study staff. The heterogeneity of the studies included in the reviews also reduced the certainty of the evidence. Subgroup analyses were often conducted on small samples and may not have achieved sufficient statistical power to detect effects and be included in this review. Another limitation was the inadequate description of the usual care treatment. For example, some studies just stated that participants in the control group received usual care but did not describe what usual care consisted of. Therefore, it was not possible to compare usual care, as assumed in the studies, with usual care in Sudan.

Our review only included studies of moderate to high quality reviews and moderate to high certainty of outcomes evidence. The results can contribute to the future design and evaluation of care transition interventions in low-income countries.

## 5. Conclusion

The study recommends that interventions to improve the transition of care from hospital to home include a combination of two, three, or four of the following components: home visits, use of information and communication technology (ICT), case manager models, multidisciplinary teams, and self-management support. The relevance of these interventions for Sudan was found to be undermined by contextual factors such as lack of human resources, telecommunications instability, and inequality in accessibility. Future studies should investigate the contextual factors that influence implementation, especially the socioeconomic and educational situations, as well as factors related to resources in terms of the health workforce and technologies. Further implementation studies are required.

## Data Availability

The original contributions presented in the study are included in the article/**Supplementary Material**, further inquiries can be directed to the corresponding author.
