# Supplementary material for: Effectiveness of hospital-to-home transitional care interventions and consultation for implementation in Sudan: a scoping review of systematic reviews
Source: Front Health Serv. 2023 Dec 14;3:1288575. doi: 10.3389/frhs.2023.1288575 (PMC10755884; doi:10.3389/frhs.2023.1288575)
Supplement: Supplementary file 1 [file Datasheet1.docx]

Effectiveness of hospital-to-home transitional care interventions and consultation for implementation in Sudan: A scoping review of systematic reviews

Asma MohamedSharif, Mohammed Elfeaki, Rayan Bushra, Armin Gemperli

*** Correspondence:**Asma Mohamedsharif
[asma.mohamedsharif@unilu.ch](mailto:asma.mohamedsharif@unilu.ch)

**Supplementary material 1:** Preliminary search terms

1. **PubMed search term**

**#1 AND #2 AND #3 AND #4 AND #4 NOT #5 NOT #6**

|  | **Concept 1**  **#1** | **Concept 2**  **#2** | **Context**  **#3** | **Inclusion of eligible study design**  **#4** | **Exclusion of non-eligible study design**  **#5** | **Population:**  **Exclusion of children**  **#6** |
| --- | --- | --- | --- | --- | --- | --- |
| **Key concepts** | Model* [tw]OR | AND Transition* [tw]OR | AND Discharg* [tw]OR | AND  Systematic review or meta analysis | Descriptive studies | Children |
| **Free text terms / natural language terms**  **(synonyms, UK/US terminology, medical/laymen’s terms, acronyms/abbreviations, drug brands, more narrow search terms)**  **Consider: phrase searching, proximity operators, truncation, wildcards, field qualification (e.g. textword)** | Intervention* [tw]OR Experi*[tw]OR Practice[tw]OR Stratg* [tw]OR Approach* [tw]OR Method* [tw]OR Program* [tw]OR Implement* [tw]OR  Progress* [tw]OR Chang* [tw]OR  Develop* [tw]OR | Transfer* [tw] * [tw]OR  "Transition of healthcare*" [tw]OR  "Transition* [tw] of health care" OR | "Post hospital*" [tw] OR  Posthospital* [tw] OR  "Home care *" [tw] OR |  |  |  |
| **Controlled vocabulary terms / Subject terms**  **(MeSH terms, Emtree terms)**  **Consider: explode, major headings, subheadings** | "Models, Nursing"[Mesh]OR "Life Change Events"[Mesh]OR "Culturally Competent Care"[Majr:NoExp]OR "Models, Educational"[Mesh]OR "Person-Centered Psychotherapy"[Majr]OR "Evidence-Based Practice"[Majr]OR "Program Development"[Majr]OR "Program Evaluation"[Majr]OR "Nursing Evaluation Research"[Majr] OR "Preventive Health Services"[Majr:NoExp]OR "Clinical Governance"[Majr:NoExp]OR  "Community Health Planning"[Majr]OR  "Growth and Development"[Majr:NoExp] | "Patient Transfer"[Mesh]OR  "Transitional Care"[Mesh]OR  "Continuity of Patient Care"[Mesh]OR | "Patient Discharge"[Mesh] OR  "Patient Discharge Summaries"[Mesh] OR  "Home Nursing"[Mesh] | "Systematic Reviews as Topic"[Mesh]OR "Meta-Analysis as Topic"[Mesh] OR "Meta-Analysis" [Publication Type] Or “Systematic Review*” |  |  |
| **Building block** | Model* [tw]OR Intervention* [tw]OR Experi*[tw]OR Practice[tw]OR Stratg* [tw]OR Approach* [tw]OR Method* [tw]OR Program* [tw]OR Implement* [tw]OR "Models, Nursing"[Mesh]OR "Life Change Events"[Mesh]OR "Culturally Competent Care"[Majr:NoExp]OR "Models, Educational"[Mesh]OR "Person-Centered Psychotherapy"[Majr]OR "Evidence-Based Practice"[Majr]OR "Program Development"[Majr]OR "Program Evaluation"[Majr]OR "Nursing Evaluation Research"[Majr] OR "Preventive Health Services"[Majr:NoExp] | Transition* [tw]OR Transfer* [tw] OR "Healthcare transition" [tw]OR "Transition health care"[tw] OR "Patient Transfer"[Mesh]OR "Transitional Care"[Mesh]OR "Continuity of Patient Care"[Mesh] | Discharg* [tw]OR "Post hospital*" [tw]OR Posthospital* [tw] OR "Home care *" [tw] OR "Patient Discharge"[Mesh] OR "Patient Discharge Summaries"[Mesh] OR "Home Nursing"[Mesh] OR "Discharge document" [tw] OR "Standardized discharge" [tw] OR "discharge summary" [tw] OR "discharge checklist" [tw] OR "discharge protocol" [tw] OR "care coordination" [tw]OR "care management" [tw] OR "telephone call" [tw] OR "home visit" [tw] OR "patient education" [tw] OR "patient involvement" [tw] OR "patient engagement" [tw] OR "family engagement" [tw] OR "information sharing" [tw] OR "patient advocate" [tw] OR "family advocate" [tw] OR "Patient Discharge Summaries"[Mesh] OR "Disease Management"[Mesh] OR "Case Managers"[Mesh]OR "House Calls"[Mesh]OR "Shared Medical Appointments"[Mesh] OR "Patient Education as Topic"[Mesh] OR "Models, Educational"[Mesh]OR "Patient Participation"[Mesh]OR "Interdisciplinary Communication"[Mesh]OR "Patient Care Team"[Mesh] OR "Patient Readmission"[Mesh] OR readmission [tw] or rehospitalization [tw] or readmittance [tw] or re-hospitalization [tw] or re-admittance [tw] or re-admission [tw] | “Systematic Review*” [tw] OR “review*” [tw] OR “Meta analysis” [tw] OR "Systematic Review" [Publication Type] OR "Systematic Reviews as Topic"[Mesh]OR "Meta-Analysis as Topic"[Mesh] OR "Meta-Analysis" [Publication Type] Or “Systematic Review*” [title] OR “review*” [title] OR “Meta analysis” [title] | “qualitative” [Title] OR “focus group” [Title] OR “case report” [Title] OR “tooth” [Title] OR “teeth” [Title] OR “dentist” [Title] OR "Equivalence Trial"[Publication Type] OR "Adaptive Clinical Trial"[Publication Type] OR "Clinical Trial"[Publication Type] OR "Randomized Controlled Trial"[Publication Type] OR "clinical trial, phase ii"[Publication Type] OR "clinical trial, phase iii"[Publication Type] OR "clinical trial, phase iv"[Publication Type] OR "Pragmatic Clinical Trial"[Publication Type] OR "Controlled Clinical Trial"[Publication Type] | "pediatrics"[MeSH] OR "pediatrics"[Title/Abstract] OR "paediatric"[Title/Abstract] OR juvenile [Title/Abstract] OR "child"[MeSH] OR "child"[Title/Abstract] OR "children"[Title/Abstract] OR "infant"[MeSH] OR "infant"[Title/Abstract] OR “infants"[Title/Abstract] OR “toddler"[Title/Abstract] |
| **Publication period** | AND "2000/01/01"[PDAT] : "2021/03/15[PDAT] |  |  |  |  |  |

1. **CIHNAL search term**

|  | **Concept 1**  **#1** | **Concept 2**  **#2** | **Concept 3**  **#3** | **Concept 4**  **#4** | **Exclusion of children**  **#5** |
| --- | --- | --- | --- | --- | --- |
| **Key concepts** | Model | AND Transition* OR | AND Discharg* OR | AND  Systematic review or meta analysis | Children |
| **Building block** | (MH "Transtheoretical Stages of Change Model") OR (MH "Models, Theoretical") OR (MH "Models, Structural") OR (MH "Models, Psychological") OR (MH "Nursing Models, Theoretical") OR (MH "King Open Systems Model") OR (MH "Models, Statistical") OR (MH "Marker Nursing Model") OR (MH "Health Belief Model") OR (MH "Orem Self-Care Model") OR "model" OR (MH "Models, Educational") OR (MH "Theory Construction") OR (MH "Quality Management, Organizational") OR (MH "Early Intervention") OR (MH "Intervention Trials") OR (MH "Nursing Interventions") OR "Intervention" OR (MH "Experimental Studies") OR (MH "Saba Clinical Care Nursing Interventions") OR (MH "Weight Management (Iowa NIC)") OR (MH "Transfer Care (Saba CCC)") OR (MH "Teaching: Procedure-Treatment (Iowa NIC)") OR (MH "Teaching: Prescribed Medication (Iowa NIC)") OR (MH "Teaching: Prescribed Diet (Iowa NIC)") OR (MH "Teaching: Prescribed Activity-Exercise (Iowa NIC)") OR (MH "Teaching: Preoperative (Iowa NIC)") OR (MH "Teaching: Individual (Iowa NIC)") OR (MH "Teaching: Group (Iowa NIC)") OR (MH "Teaching: Disease Process (Iowa NIC)") OR (MH "Self-Responsibility Facilitation (Iowa NIC)") OR (MH "Self-Esteem Enhancement (Iowa NIC)") OR (MH "Self-Care Component (Saba CCC)") OR (MH "Self-Care Facilitation (Iowa NIC)") OR (MH "Self-Care Assistance: Toileting (Iowa NIC)") OR (MH "Self-Care Assistance: Dressing-Grooming (Iowa NIC)") OR (MH "Self-Care Assistance: Feeding (Iowa NIC)") OR (MH "Self-Care Assistance: Bathing-Hygiene (Iowa NIC)") OR (MH "Self-Care Assistance (Iowa NIC)") OR (MH "Self-Awareness Enhancement (Iowa NIC)") OR (MH "Safety Component (Saba CCC)") OR (MH "Quality Monitoring (Iowa NIC)") OR (MH "Personal Care (Saba CCC)") OR (MH "Patient Education (Iowa NIC)") OR (MH "Nursing Care Studies") OR (MH "Nursing Care Coordination (Saba CCC)") OR (MH "Nurse Specialist Service (Saba CCC)") OR (MH "Mobility Therapy (Saba CCC)") OR (MH "Mouth Care (Saba CCC)") OR (MH "Meditation (Iowa NIC)") OR (MH "Medication Treatment (Saba CCC)") OR (MH "Medication Side Effects (Saba CCC)") OR (MH "Medication Prescribing (Iowa NIC)") OR (MH "Medication Prefill Preparation (Saba CCC)") OR (MH "Medication Managements (Iowa NIC)") OR (MH "Medication Management (Iowa NIC)") OR (MH "Medication Component (Saba CCC)") OR (MH "Medication Care (Saba CCC)") OR (MH "Medication Administration: Enteral (Iowa NIC)") OR (MH "Medication Administration (Iowa NIC)") OR (MH "Medication Actions (Saba CCC)") OR (MH "Home Health Aide Service (Saba CCC)") OR (MH "Home Situation Analysis (Saba CCC)") OR (MH "Home Maintenance Assistance (Iowa NIC)") OR (MH "Hospice (Saba CCC)") OR (MH "Hope Instillation (Iowa NIC)") OR (MH "Health System Management (Iowa NIC)") OR (MH "Health System Mediation (Iowa NIC)") OR (MH "Health System Guidance (Iowa NIC)") OR (MH "Health System (Iowa NIC)") OR (MH "Fertility Care (Saba CCC)") OR (MH "Family Therapy (Iowa NIC)") OR (MH "Family Support (Iowa NIC)") OR (MH "Family Process Maintenance (Iowa NIC)") OR (MH "Family Process Analysis (Saba CCC)") OR (MH "Evaluation Research") OR (MH "Decision-Making Support (Iowa NIC)") OR (MH "Counseling (Iowa NIC)") OR (MH "Counseling Service (Saba CCC)") OR (MH "Caregiver Support (Iowa NIC)") OR (MH "Activity and Exercise Enhancement (Iowa NIC)") OR (MH "Activity Care (Saba CCC)") | ( (MH "Nursing Care Coordination (Saba CCC)") OR (MH "Multidisciplinary Care Team") OR (MH "Health Care Costs") OR (MH "Nursing Care Plans, Computerized") OR (MH "Cancer Care Facilities") OR (MH "Age Specific Care") OR (MH "Nursing Care Plans") ) OR ( Transition* OR Transfer* OR "Healthcare transition" OR "Transition health care" OR "Patient Transfer" OR "Transitional Care" OR "Continuity of Patient Care" ) | ( (MH "Patient Discharge Education") OR (MH "Transfer, Discharge") OR (MH "Early Patient Discharge") OR (MH "Discharge Planning") OR (MH "Patient Discharge Summaries") OR (MH "Patient Discharge") OR (MH "Discharge Planning (Iowa NIC)") OR (MH "Library User Education") OR (MH "Outcomes of Education") OR "discharge education or discharge instructions or discharge teaching" OR (MH "Models, Educational") OR (MH "Patient Education") OR (MH "Teaching, Guidance, and Counseling (Omaha)") ) OR ( Discharg* OR "Post hospital*" OR Posthospital* OR "Home care *" OR "Patient Discharge" OR "Patient Discharge Summaries" OR "Home Nursing" OR “Discharge document” OR “Standardized discharge” OR “discharge checklist” OR “discharge protocol” OR “care coordination” OR “telephone call” OR “home visit” OR “patient education” OR “patient involvement” OR “patient engagement” OR “family engagement” OR “information sharing” OR “patient advocate” OR “family advocate” OR "Patient Discharge Summaries" OR "Nursing, Team" OR "House Calls" OR "Comprehensive Health Care" OR "Shared Medical Appointments" OR "Patient Education as Topic"[Mesh] OR "Models, Educational" OR "Self Care" OR "Patient Participation" OR "Interdisciplinary Communication" OR "Patient Care Team" ) OR "readmission or rehospitalization or readmittance or re-hospitalization or re-admittance or re-admission" OR (MH "Readmission") | ( (MM "Systematic Review") OR (MM "Scoping Review") ) OR (MH "Meta Analysis") OR ( “Systematic Review*” OR “review*” OR “Meta analysis” ) | pediatric* OR paediatric* OR juvenile OR child OR infant OR infant OR toddler |
| **Restriction of Publication period** | AND "2000/01/01"[PDAT] : "2021/03/15[PDAT] |  |  |  |  |

1. **Cochrane search term**

#1 OR #2 OR #3 OR #4

#1: MeSH descriptor: [Transitional Care] explode all trees

#2: MeSH descriptor: [Patient Discharge] explode all trees

#3: MeSH descriptor: [Patient readmissions] explode all trees

#4: MeSH descriptor: [Medical Reconciliation] explode all trees
