# Supplementary material for: Effectiveness of hospital-to-home transitional care interventions and consultation for implementation in Sudan: a scoping review of systematic reviews
Source: Front Health Serv. 2023 Dec 14;3:1288575. doi: 10.3389/frhs.2023.1288575 (PMC10755884; doi:10.3389/frhs.2023.1288575)
Supplement: Supplementary file 2 [file Datasheet2.docx]

Supplementary Material

Effectiveness of hospital-to-home transitional care interventions and consultation for implementation in Sudan: A scoping review of systematic reviews

Asma MohamedSharif, Mohammed Elfeaki, Rayan Bushra, Armin Gemperli

*** Correspondence:**Asma Mohamedsharif
[asma.mohamedsharif@unilu.ch](mailto:asma.mohamedsharif@unilu.ch)

**Supplementary Material 2: Quality assessment of the selected papers using AMSTAR-2**

| **Authors** | **Item.1** |  | **Item.2** | **Item.3** | **Item.4** | **Item.5** | **Item.6** | **Item.7** | **Item.8** | **Item.9** | **Item.10** | **Item. 11** | **Item. 12** | **Item.13** | **Item.14** | **Item. 15** | **Item.16** |
| --- | --- | --- | --- | --- | --- | --- | --- | --- | --- | --- | --- | --- | --- | --- | --- | --- | --- |
| **Verloo H. et al. 2017** | Yes |  | No | No | Yes | Yes | Yes | Yes | Yes | Yes | No | No Meta-analysis conducted | No Meta-analysis conducted | No | Yes | No meta-analysis conducted | Yes |
| **Holzinger F. et al. 2017** | Yes |  | Yes | Yes | Yes | Yes | Yes | Yes | Yes | Includes only RCTs or NRSI | Yes | Yes | Yes | Yes | Yes | Yes | Yes |
| **Mia E. et al. 2020** | Yes |  | Yes | Yes | Yes | Yes | Yes | Yes | Partial Yes | Includes only RCTs or NRSI | No | Yes | No | No | Yes | No | Yes |
| **Linertová R. et al. 2010** | Yes |  | No | No | Partial Yes | Yes | Yes | Yes | Partial Yes | No | No | No Meta-analysis conducted | No Meta-analysis conducted | No | Yes | No meta-analysis conducted | Yes |
| **Villeneuve Y. et al. 2021** | No |  | No | Yes | No | Yes | Yes | Yes | Partial Yes | No | No | No Meta-analysis conducted | No Meta-analysis conducted | No | No | No meta-analysis conducted | Yes |
| **Janice L. et al. 2013** | Yes |  | No | Yes | No | Yes | Yes | Partial Yes | Partial Yes | Partial Yes | No | No | No Meta-analysis conducted | No | No | No | Yes |
| **Domingo Grace R. et al. 2012** | Yes |  | Yes | Yes | Partial Yes | No | No | Yes | Partial Yes | No | Yes | No Meta-analysis conducted | No Meta-analysis conducted | No | Yes | No meta-analysis conducted | No |
| **Braet A. et al 2016** | Yes |  | Yes | No | Yes | Yes | Yes | Yes | Yes | Yes | No | Yes | No Meta-analysis conducted | Yes | Yes | Yes | Yes |
| **Feltner C. et al. 2014** | Yes |  | Yes | No | No | Yes | Yes | Partial Yes | Partial Yes | No | No | Yes | Yes | Yes | Yes | No | Yes |
| **Van Spall H.G.C et al. 2017** | Yes |  | Yes | No | Partial Yes | Yes | Yes | Yes | Partial Yes | Partial Yes | Yes | Yes | Yes | No | Yes | No | Yes |
| **Facchinettia G. et al. 2020** | Yes |  | Yes | No | Yes | Yes | Yes | Yes | Yes | Yes | No | Yes | Yes | Yes | Yes | Yes | Yes |
| **Vedel and Khanasso 2015** | Yes |  | Yes | No | Partial Yes | Yes | Yes | Partial Yes | Yes | Partial Yes | No | Yes | Yes | Yes | No | Yes | Yes |
| **Naderi C. et al 2018** | Yes |  | Yes | Yes | Yes | Yes | Yes | Partial Yes | No | Partial Yes | No | Yes | Yes | No | Yes | No | Yes |
| **Ridwan E. et al 2019** | Yes |  | No | No | Partial Yes | Yes | Yes | Yes | Yes | Yes | No | Yes | Yes | No | Yes | Yes | Yes |
| **Morkisch N. et al 2020** | Yes |  | Yes | No | Partial Yes | Yes | No | Partial Yes | Partial Yes | Partial Yes | Yes | No Meta-analysis conducted | No Meta-analysis conducted | Yes | No | No meta-analysis conducted | Yes |
| **Okrainec K. et al 2017** | No |  | No | No | Partial Yes | Yes | Yes | Partial Yes | Yes | Partial Yes | No | No Meta-analysis conducted | No Meta-analysis conducted | Yes | Yes | No meta-analysis conducted | Yes |
| **Le Berre M. et al 2017** | Yes |  | Yes | No | Yes | Yes | Yes | No | No | Yes | No | Yes | Yes | Yes | Yes | No meta-analysis conducted | Yes |
| **Kim H. et al 2015** | Yes |  | No | No | Partial Yes | No | No | No | Partial Yes | No | No | No Meta-analysis conducted | No Meta-analysis conducted | No | No | No meta-analysis conducted | Yes |
| **Branowicki P. et al 2016** | Yes |  | Partial Yes | No | Partial Yes | Yes | Yes | Yes | Partial Yes | Yes | No | No | Yes | No | Yes | No | Yes |
| **Yang F et al 2017** | Yes |  | No | No | No | Yes | Yes | Yes | Yes | No | No | Yes | Yes | No | Yes | Yes | Yes |
| **Bonetti A. et al 2020** | Yes |  | Yes | No | Yes | Yes | Yes | Yes | Yes | Partial Yes | Yes | Yes | Yes | Yes | Yes | Yes | No |
| **Uminski K. et al 2018** | Yes |  | Partial Yes | Yes | Partial Yes | Yes | Yes | Partial Yes | Partial Yes | Partial Yes | No | Yes | Yes | Yes | Yes | Yes | Yes |
| **Wang Y. et al 2007** | Yes |  | No | No | Partial Yes | Yes | Yes | Yes | Partial Yes | Partial Yes | No | Yes | Yes | No | Yes | Yes | Yes |
| **Li Y. et al 2020** | Yes |  | Partial Yes | No | Partial Yes | Yes | Yes | Partial Yes | Partial Yes | Partial Yes | No | Yes | Yes | Yes | Yes | Yes | Yes |
| **Echevarria C. et al 2016** | Yes |  | No | No | Partial Yes | Yes | No | Partial Yes | Partial Yes | Partial Yes | No | Yes | Yes | Yes | Yes | Yes | Yes |
| **Chiu W. et al 2007** | Yes |  | No | No | No | No | No | Partial Yes | Partial Yes | No | No | No | No Meta-analysis conducted | No | No | No | Yes |
| **Allen J. et al 2014** | Yes |  | No | No | Partial Yes | Yes | No | Yes | Partial Yes | Partial Yes | No | No Meta-analysis conducted | No Meta-analysis conducted | No | Yes | No meta-analysis conducted | Yes |
| **Majothi D. et al 2015** | Yes |  | Partial Yes | No | Yes | Yes | Yes | No | Partial Yes | Partial Yes | No | Yes | Yes | No | Yes | No | Yes |
| **Ma D. et al 2018** | Yes |  | No | No | Yes | Yes | Yes | Partial Yes | Partial Yes | Partial Yes | No | Yes | Yes | No | Yes | No | Yes |
| **T. Munk et al 2015** | Yes |  | No | No | Partial Yes | Yes | Yes | Partial Yes | Partial Yes | Partial Yes | No | Yes | Yes | Yes | Yes | No | Yes |
| **Zhu QM. et al 2015** | Yes |  | No | No | No | No | Yes | Partial Yes | Partial Yes | Partial Yes | No | Yes | Yes | No | Yes | Yes | Yes |
| **Langhorne. P et al 2005** | Yes |  | No | No | Partial Yes | Yes | No | Partial Yes | Partial Yes | No | No | Yes | No | No | Yes | No | Yes |
| **Lambrinou E. et al 2011** | Yes |  | No | No | Partial Yes | Yes | Yes | Partial Yes | Partial Yes | Partial Yes | No | Yes | No | No | Yes | Yes | Yes |
| **Oliveira G. et al 2017** | Yes |  | No | No | Partial Yes | Yes | Yes | Partial Yes | Partial Yes | Partial Yes | No | Yes | Yes | No | Yes | Yes | Yes |
| **Phillips C. et al 2004** | Yes |  | No | No | Partial Yes | No | Yes | Partial Yes | Partial Yes | Partial Yes | No | Yes | Yes | Yes | Yes | Yes | Yes |
| **O’Donnell R. et al 2020** | Yes |  | No | No | Partial Yes | Yes | Yes | Partial Yes | Partial Yes | Partial Yes | No | No Meta-analysis conducted | No Meta-analysis conducted | No | Yes | No meta-analysis conducted | Yes |
| **Garcia M. et al 2019** | Yes |  | No | No | No | No | Yes | Partial Yes | Partial Yes | No | No | No | No Meta-analysis conducted | No | Yes | No | Yes |
| **Slyer J. et al 2011** | Yes |  | No | No | Partial Yes | Yes | No | No | No | Partial Yes | No | Yes | No | Yes | Yes | No | Yes |
| **Lee J. et al 2010** | Yes |  | No | Yes | Yes | Yes | Yes | No | Partial Yes | Partial Yes | No | Yes | No | No | Yes | No | Yes |
| **Penney L. et al 2018** | Yes |  | No | Yes | No | Yes | Yes | Partial Yes | Partial Yes | No | No | No Meta-analysis conducted | No Meta-analysis conducted | No | No | No meta-analysis conducted | Yes |
| **Pritchard E. et al 2014** | Yes |  | No | No | Yes | Yes | Yes | Partial Yes | Partial Yes | Partial Yes | Yes | Yes | Yes | No | Yes | No | Yes |
| **Parker SG. et al 2002** | Yes |  | No | No | Partial Yes | Yes | Yes | No | Partial Yes | No | No | No Meta-analysis conducted | No Meta-analysis conducted | No | Yes | No meta-analysis conducted | Yes |
| **Richards S. et al 2003** | Yes |  | No | No | Partial Yes | Yes | Yes | Yes | Partial Yes | No | Yes | No Meta-analysis conducted | No Meta-analysis conducted | No | Yes | No meta-analysis conducted | No |
| **Bethishou L. et al 2019** | Yes |  | No | No | No | Yes | Yes | Partial Yes | Partial Yes | Partial Yes | No | No Meta-analysis conducted | No Meta-analysis conducted | Yes | Yes | No meta-analysis conducted | Yes |
| **Bakhshayesh S. et al 2020** | Yes |  | No | Yes | Partial Yes | No | No | Partial Yes | Yes | Partial Yes | Yes | Yes | Yes | No | Yes | Yes | Yes |
| **Piraino E. et al 2012** | Yes |  | No | No | Partial Yes | Yes | No | No | Partial Yes | No | No | No Meta-analysis conducted | No Meta-analysis conducted | No | No | No meta-analysis conducted | No |
| **Conroy S. et al 2011** | Yes |  | No | No | Partial Yes | Yes | No | Partial Yes | Partial Yes | No | No | Yes | Yes | No | Yes | Yes | Yes |
| **Pedersen P. et al 2017** | Yes |  | Partial Yes | No | Partial Yes | No | Yes | Partial Yes | Partial Yes | Partial Yes | No | Yes | Yes | Yes | Yes | No | Yes |
| **Spinewine A. et al 2013** | No |  | No | No | Partial Yes | Yes | Yes | Partial Yes | Partial Yes | Partial Yes | No | No Meta-analysis conducted | No Meta-analysis conducted | Yes | No | No meta-analysis conducted | Yes |
| **Lina S. et al 2019** | Yes |  | Partial Yes | No | Partial Yes | Yes | No | Partial Yes | Partial Yes | Partial Yes | No | Yes | Yes | No | Yes | Yes | Yes |
| **Fox M. et al 2013** | Yes |  | No | No | Partial Yes | Yes | Yes | Partial Yes | Partial Yes | Partial Yes | Yes | Yes | Yes | No | Yes | No | Yes |
| **Redmond P. et al 2018** | Yes |  | Yes | No | Yes | Yes | Yes | Partial Yes | Yes | Partial Yes | No | Yes | Yes | Yes | Yes | No | Yes |
| **Griiths PD. et al 2009** | Yes |  | No | No | Partial Yes | No | Yes | Partial Yes | Partial Yes | Partial Yes | No | Yes | Yes | No | Yes | No | Yes |
| **Johnson A. et al 2010** | Yes |  | No | No | Partial Yes | No | Yes | No | Partial Yes | Partial Yes | No | No Meta-analysis conducted | No Meta-analysis conducted | Yes | No | No meta-analysis conducted | Yes |
| **Bradley G. et al 2016** | Yes |  | Partial Yes | No | Partial Yes | Yes | Yes | Partial Yes | Yes | Partial Yes | Yes | No Meta-analysis conducted | No Meta-analysis conducted | Yes | Yes | No | Yes |
| **Mistiaen and Poot . et al 2006** | Yes |  | Partial Yes | No | Partial Yes | Yes | Yes | Partial Yes | Partial Yes | Partial Yes | Yes | Yes | Yes | Yes | Yes | Yes | Yes |
| **Takeda A. et al 2019** | Yes |  | Partial Yes | No | Partial Yes | Yes | Yes | Partial Yes | Yes | Partial Yes | Yes | Yes | Yes | Yes | Yes | Yes | Yes |

**Item.1:** Did the research questions and inclusion criteria for the review include the components of PICO?

**Item.2:** Did the report of the review contain an explicit statement that the review methods were established prior to the conduct of the review and did the report justify any significant deviations from the protocol?

**Item.3:** Did the review authors explain their selection of the study designs for inclusion in the review?

**Item.4:** Did the review authors use a comprehensive literature search strategy?

**Item.5:** Did the review authors perform study selection in duplicate?

**Item.6:** Did the review authors perform data extraction in duplicate?

**Item.7:** Did the review authors provide a list of excluded studies and justify the exclusions?

**Item.8:** Did the review authors describe the included studies in adequate detail?

**Item.9:** Did the review authors use a satisfactory technique for assessing the risk of bias (RoB) in individual studies that were included in the review?

**Item.10:** Did the review authors report on the sources of funding for the studies included in the review?

**Item.11:** If meta-analysis was performed did the review authors use appropriate methods for statistical combination of results?

**Item.12:** If meta-analysis was performed, did the review authors assess the potential impact of RoB in individual studies on the results of the meta-analysis or other evidence synthesis?

**Item.13:** Did the review authors account for RoB in individual studies when interpreting/ discussing the results of the review?

**Item.14:** Did the review authors provide a satisfactory explanation for, and discussion of, any heterogeneity observed in the results of the review?

**Item.15:** If they performed quantitative synthesis did the review authors carry out an adequate investigation of publication bias (small study bias) and discuss its likely impact on the results of the review?

**Item.16:** Did the review authors report any potential sources of conflict of interest, including any funding they received for conducting the review?
