# Supplementary material for: Effectiveness of hospital-to-home transitional care interventions and consultation for implementation in Sudan: a scoping review of systematic reviews
Source: Front Health Serv. 2023 Dec 14;3:1288575. doi: 10.3389/frhs.2023.1288575 (PMC10755884; doi:10.3389/frhs.2023.1288575)
Supplement: Supplementary file 3 [file Datasheet3.docx]

Supplementary Material

Effectiveness of hospital-to-home transitional care interventions and consultation for implementation in Sudan: A scoping review of systematic reviews

Asma MohamedSharif, Mohammed Elfeaki, Rayan Bushra, Armin Gemperli

*** Correspondence:**Asma Mohamedsharif
[asma.mohamedsharif@unilu.ch](mailto:asma.mohamedsharif@unilu.ch)

**Supplementary material 3: Summary of Finding (SoF) tables**

| **S1 Table: Braet A et al., 2016: discharge interventions compared to usual care for a health problem and/or population** | | | | | |
| --- | --- | --- | --- | --- | --- |
| **Patient or population:** health problem and/or population  **Setting:** hospital to home  **Intervention:** discharge interventions  **Comparison:** usual care | | | | | |
| Outcomes | **Anticipated absolute effects^*^** (95% CI) | | Relative effect (95% CI) | № of participants (studies) | Certainty of the evidence (GRADE) |
|  | **Risk with usual care** | **Risk with discharge interventions** |  |  |  |
| Readmission | 174 per 1,000 | **134 per 1,000** (134 to 146) | **RR 0.77** (0.77 to 0.84) | 16242 (47 RCTs) | ⨁◯◯◯ Very low |
| ED visits | The overall effect was not statistically significant (RR, 0.75 [95% CI, 0.55-1.01]; p=0.06) | |  | (10 RCTs) | ⨁◯◯◯ Very low |
| Mortality | The discharge interventions had no overall effect on mortality (RR, 0.70 [95% CI, 0.48-1.01]; p=0.06) | |  | (14 RCTs) | ⨁◯◯◯ Very low |
| Quality of life | - | | | | Not reported |
| Cost | - | | | | Not reported |
| Adverse events | - | | | | Not reported |

| **S2 Table: Takeda, et al., 2019: clinical-based interventions compared to the usual care for heart failure** | | | | | |
| --- | --- | --- | --- | --- | --- |
| **Patient or population:** adult with heart failure  **Setting:** heart-failure clinic (outpatients, community)  **Intervention:** heart-failure clinic  **Comparison:** usual care | | | | | |
| Outcomes | **Anticipated absolute effects^*^** (95% CI) | | Relative effect (95% CI) | № of participants (studies) | Certainty of the evidence (GRADE) |
|  | **Risk with usual care** | **Risk with heart-failure clinic** |  |  |  |
| Heart-failure-related hospital readmission | 345 per 1,000 | 348 per 1,000 | **RR 1.01** (0.87 to 1.18) | 887  (2 RCTs) | ⨁⨁⨁◯ Moderate |
| All-causes hospital readmission | 273 per 1,000 | 238 per 1,000 | **RR 0.87** (0.68 to 1.10) | 1129  (4 RCTs) | ⨁⨁◯◯ Low |
| All-cause mortality | 273 per 1,000 | 238 per 1,000 | **RR 0.87** (0.68 to 1.10) | 1686  (7 RCTs) | ⨁⨁◯◯ Low |
| Quality of life | 1 study reported no difference in MLHFQ between groups at 1 year, and another reported similar changes from baseline for both intervention and control groups. 2 studies used the Nottingham Health Profile (NHP) rather than the MLHFQ, both reporting similar scores in intervention and control groups. | | | 641  (4 RCTs) | ⨁⨁◯◯ Low |
| Cost | 1 study reported a cost saving of EUR 1382 per person, the other saving of USD 1300 per person | | | 390  (2 RCTs) | ⨁⨁◯◯ Low |
| Adverse events | - | | | | Not reported |

| **S3 Table: Takeda, et al., 2019: multidisciplinary disease management programs compared to usual care for heart failure** | | | | | |
| --- | --- | --- | --- | --- | --- |
| **Patient or population:** adult with heart failure  **Setting:** community  **Intervention:** multidisciplinary disease management programs  **Comparison:** usual care | | | | | |
| Outcomes | **Anticipated absolute effects^*^** (95% CI) | | Relative effect (95% CI) | № of participants (studies) | Certainty of the evidence (GRADE) |
|  | **Risk with usual care** | **Risk with heart-failure clinic** |  |  |  |
| Heart-failure-related hospital readmission | 159 per 1000 | 197 per 1000 | **RR 0.68** (0.50 to 0.92) | 1108  (5 RCTs) | ⨁⨁◯◯ Low |
| All-causes hospital readmission | 450 per 1,000 | 383 per 1,000 | **RR 0.85** (0.71 to 1.01) | 1152  (5 RCTs) | ⨁⨁◯◯ Low |
| Heart-failure-related mortality | 159 per 1000 | 73 per 1000 | **RR 0.46** (0.23 to 0.95) | 277  (2 RCTs) | ⨁◯◯◯ Very low |
| All-cause mortality | 185 per 1,000 | 124 per 1,000 | **RR 0.67** (0.54 to 0.83) | 1764  (8 RCTs) | ⨁⨁⨁◯ Moderate |
| Quality of life | 1 study reported a score at tend of the follow-up (34.3 in the usual care group)  1 study reported a decrease from the baseline of 0.5 in the usual care group | | | 140  (2 RCTs) | ⨁◯◯◯ Very low |
| Cost | Only 1 multidisciplinary intervention study reported cost, the cost per addition management program compared with usual care was EUR 38,274 from a healthcare perspective and EUR 25,390 from a societal perspective | | | 117  (1 RCT) | ⨁⨁◯◯ Low |
| Adverse events | - | | | | Not reported |

| **S4 Table: Takeda, et al., 2019: case management compared to usual care for heart failure** | | | | | |
| --- | --- | --- | --- | --- | --- |
| **Patient or population:** adult with heart failure  **Setting:** community  **Intervention:** case management  **Comparison:** usual care | | | | | |
| Outcomes | **Anticipated absolute effects^*^** (95% CI) | | Relative effect (95% CI) | № of participants (studies) | Certainty of the evidence (GRADE) |
|  | **Risk with usual care** | **Risk with heart-failure clinic** |  |  |  |
| Heart-failure-related hospital readmission | 361 per 1000 | 231 per 1000 | **RR 0.64** (0.53 to 0.78) | 2528  (12 RCTs) | ⨁⨁⨁◯ Moderate |
| All-causes hospital readmission | 491 per 1,000 | 451 per 1,000 | **RR 0.92** (0.83 to 1.01) | 4539  (14 RCTs) | ⨁⨁⨁◯ Moderate |
| All-cause mortality | 187 per 1,000 | 146 per 1,000 | **RR 0.78** (0.68 to 0.90) | 6903  (26 RCTs) | ⨁⨁◯◯ Low |
| Quality of life | Six studies show a broadly positive effect of case management interventions; however, two small studies indicate that QoL may actually be lower in the case management groups. | | | 1595  (8 RCTs) | ⨁◯◯◯ Very low |
| Coast | Case management studies reported the costs of their programs, although the wide range in dates and locations of studies complicates interpretation: 2 reported higher costs for intervention groups; 4 reported lower costs (generally after taking readmission costs into account); and 1 reported no difference in costs | | | 117  (1 RCTs) | ⨁⨁◯◯ Low |
| Adverse events | - | | | | Not reported |

| **S5 Table: Li Y et al., 2021: transitional care interventions compared to usual care for patients with heart failure** | | | | | |
| --- | --- | --- | --- | --- | --- |
| **Patient or population:** Patients with Heart Failure  **Setting:** Hospital to home  **Intervention:** Transitional Care Interventions  **Comparison:** usual care | | | | | |
| Outcomes | **Anticipated absolute effects^*^** (95% CI) | | Relative effect (95% CI) | № of participants (studies) | Certainty of the evidence (GRADE) |
|  | **Risk with usual care** | **Risk with transitional Care Interventions** |  |  |  |
| Heart-failure-related hospital readmission | - | | **RR 0.78** (0.68 to 0.89) | (17 RCTs) | ⨁⨁⨁⨁ High |
| All-cause hospital readmission | - | | **RR 0.89** (0.82 to 0.97) | (29 RCTs) | ⨁⨁◯◯ Low |
| ED visit | - | | **RR 0.94** (0.83 to 1.07) | (6 RCTs) | ⨁⨁⨁◯ Moderate |
| Mortality | - | | | | Not reported |
| Quality of life | - | | | | Not reported |
| Cost | - | | | | Not reported |
| Adverse events | - | | | | Not reported |

| **S6 Table: Vedel and Khanassov, 2015: transitional care compared to usual care for patients with congestive heart failure** | | | | | |
| --- | --- | --- | --- | --- | --- |
| **Patient or population:** patients with congestive heart failure  **Setting**: hospital to home  **Intervention**: transitional care  **Comparison**: usual care | | | | | |
| Outcomes | **Anticipated absolute effects^*^** (95% CI) | | Relative effect (95% CI) | № of participants (studies) | Certainty of the evidence (GRADE) |
|  | **Risk with usual care** | **Risk with Transitional Care** |  |  |  |
| All-cause hospital readmissions | 478 per 1,000 | **440 per 1,000** (416 to 469) | **RR 0.92** (0.87 to 0.98) | 10863 (43 RCTs) | ⨁⨁◯◯ Low |
| All-cause ed visits | 453 per 1,000 | **322 per 1,000** (236 to 444) | **RR 0.71** (0.52 to 0.98) | 808 (5 RCTs) | ⨁◯◯◯ Very low |
| Mortality | - | | | | Not reported |
| Quality of life | - | | | | Not reported |
| Cost | - | | | | Not reported |
| Adverse events | - | | | | Not reported |

| **S7 Table: Uminski K et al., 2018: post-discharge virtual wards compared to usual care for heart failure populations** | | | | | |
| --- | --- | --- | --- | --- | --- |
| **Patient or population:** heart failure populations  **Setting:** Hospital to home  **Intervention** post-discharge virtual wards  **Comparison:** usual care | | | | | |
| Outcomes | **Anticipated absolute effects^*^** (95% CI) | | Relative effect (95% CI) | № of participants (studies) | Certainty of the evidence (GRADE) |
|  | **Risk with usual care** | **Risk with post-discharge virtual wards** |  |  |  |
| Heart-failure-related hospital readmission | 270 per 1,000 | **162 per 1,000** (132 to 205) | **RR 0.60** (0.49 to 0.76) | 1510 (6 RCTs) | ⨁⨁⨁◯ Moderate |
| All-cause hospital readmission | 428 per 1,000 | **368 per 1,000** (287 to 475) | **RR 0.86** (0.67 to 1.11) | 1634 (6 RCTs) | ⨁◯◯◯ Very low |
| All-cause mortality | 146 per 1,000 | **86 per 1,000** (64 to 114) | **RR 0.59** (0.44 to 0.78) | 1634 (6 RCTs) | ⨁⨁⨁◯  Moderate |
| Quality of life | - | | | | Not reported |
| Cost | - | | | | Not reported |
| Adverse events | - | | | | Not reported |

| **S8 Table: Uminski K et al., 2018: post-discharge virtual wards compared to usual care for heart failure populations** | | | | | |
| --- | --- | --- | --- | --- | --- |
| **Patient or population:** heart failure populations  **Setting:** hospital to home  **Intervention** post-discharge virtual wards  **Comparison:** usual care | | | | | |
| Outcomes | **Anticipated absolute effects^*^** (95% CI) | | Relative effect (95% CI) | № of participants (studies) | Certainty of the evidence (GRADE) |
|  | **Risk with usual care** | **Risk with post-discharge virtual wards** |  |  |  |
| Heart-failure-related hospital readmission | 270 per 1,000 | **162 per 1,000** (132 to 205) | **RR 0.60** (0.49 to 0.76) | 1510 (6 RCTs) | ⨁⨁◯◯ Low |
| All-cause hospital readmission | 428 per 1,000 | **368 per 1,000** (287 to 475) | **RR 0.86** (0.67 to 1.11) | 1634 (6 RCTs) | ⨁◯◯◯ Very low |
| All-cause mortality | 146 per 1,000 | **86 per 1,000** (64 to 114) | **RR 0.59** (0.44 to 0.78) | 1634 (6 RCTs) | ⨁⨁◯◯ Low |
| Quality of life | - | | | | Not reported |
| Cost | - | | | | Not reported |
| Adverse events | - | | | | Not reported |

| **S9 Table : Facchinetti G et al., 2020: continuity of care interventions compared to usual care for older people with chronic diseases** | | | | | |
| --- | --- | --- | --- | --- | --- |
| **Patient or population:** older people with chronic diseases  **Setting:** hospital to home  **Intervention:** continuity of care interventions  **Comparison:** usual care | | | | | |
| Outcomes | **Anticipated absolute effects^*^** (95% CI) | | Relative effect (95% CI) | № of participants (studies) | Certainty of the evidence (GRADE) |
|  | **Risk with usual care** | **Risk with Continuity of care interventions** |  |  |  |
| Readmission =1 month | 160 per 1,000 | **135 per 1,000** (114 to 159) | **RR 0.84** (0.71 to 0.99) | 3240 (10 RCTs) | ⨁⨁⨁⨁ High |
| Readmission = 3 months | 299 per 1,000 | **221 per 1,000** (194 to 251) | **RR 0.74** (0.65 to 0.84) | 3003 (11 RCTs) | ⨁⨁⨁⨁ High |
| Readmission 3-6 months | 415 per 1,000 | **377 per 1,000** (323 to 440) | **RR 0.91** (0.78 to 1.06) | 4225 (11 RCTs) | ⨁◯◯◯ Very low |
| Readmission 6- 12 months | 361 per 1,000 | **303 per 1,000** (267 to 342) | **RR 0.84** (0.74 to 0.95) | 4032 (13 RCTs) | ⨁◯◯◯ Very low |
| Mortality | - | | | | Not reported |
| Quality of life | - | | | | Not reported |
| Cost | - | | | | Not reported |
| Adverse events | - | | | | Not reported |

| **S10 Table: Morkisch N et al., 2020: highly intensive intervention with a total score of 28 out of 31 points compared to usual care for geriatric with heart failure and comorbidities e.g. hypertension or diabetes** | | | |
| --- | --- | --- | --- |
| **Patient or population:** geriatric with heart failure and comorbidities e.g. hypertension or diabetes  **Setting:** hospital to home  **Intervention**: highly intensive intervention with a total score of 28 out of 31 points  **Comparison:** usual care | | | |
| Outcomes | Impact | № of participants (studies) | Certainty of the evidence (GRADE) |
|  |  |  |  |
| Readmission | There is a significant difference in the readmission rate of the participants from the control (42.1%) compared with the participants from the intervention group (28.9%), p = 0.03) | (1 RCT) | ⨁⨁⨁◯ Moderate |
| Mortality | There is no significant difference, but there are differences in the percentage of deaths of the control group (12.1%) vs the intervention group (9.2%) | (1 RCT) | ⨁⨁⨁◯ Moderate |
| Quality of life | Rich and colleagues observed a statistically significant improvement of this construct in the intervention group compared to the control group (p = 0.001) | (1 RCT) | ⨁⨁⨁◯ Moderate |
| Cost | The study showed $460 less per patient | (1 RCT) | ⨁⨁⨁◯ Moderate |
| Adverse event | - | | Not reported |

| **S11 Table: Morkisch N et al., 2020: moderate intensive intervention with a total score of 16 and 18 out of 31 points compared to usual care for geriatric with heart failure or any other disease** | | | |
| --- | --- | --- | --- |
| **Patient or population:** geriatric with heart failure or any other disease  **Setting:** hospital to home  **Intervention:** moderate intensive intervention with a total score of 16 and 18 out of 31 points. (2,3)  **Comparison:** usual care | | | |
| Outcomes | Impact | № of participants (studies) | Certainty of the evidence (GRADE) |
|  |  |  |  |
| Readmission | One study found no statistically significant differences between the percent of readmission (3 months of both groups at three months of follow-up were reported. The other one showed a significate difference in the short term at 2 months (Percent differences =−54.4, p= < 0.05), 6 months (Percent differences =−42.4, P= < 0.05), and no significance at long term 12 months. | (2 RCTs) | ⨁⨁⨁◯ Moderate |
| Mortality | Significant differences between the percentage of deaths of the control (29.7%) vs intervention (12.9%) group for the period of 12 months of follow-up (p = 0.017) | (2 RCTs) | ⨁⨁⨁◯ Moderate |
| Quality of life | They didn’t observe statistically significant in the intervention group compared to the control | (2 RCTs) | ⨁⨁◯◯ Low |
| Cost | The intervention group evidenced savings of €578 per patient. | (2 RCTs) | ⨁⨁◯◯ Low |
| Adverse event | - | | Not reported |

| **S12 Table: Bonetti A et al., 2020: impact of pharmacist-led discharge counseling compared to usual care for patients** | | | | | |
| --- | --- | --- | --- | --- | --- |
| **Patient or population:** patients  **Setting:** hospital to home  **Intervention:** impact of pharmacist-led discharge counseling  **Comparison:** usual care | | | | | |
| Outcomes | **Anticipated absolute effects^*^** (95% CI) | | Relative effect (95% CI) | № of participants (studies) | Certainty of the evidence (GRADE) |
|  | **Risk with usual care** | **Risk with pharmacist-led Discharge Counseling** |  |  |  |
| Readmission | - | | **RR 0.86** (0.76 to 0.99) | (18 RCTs) | ⨁◯◯◯ Very low |
| ED Visit | - | | **RR 0.70**  (0.54 to 0.91) | (8 RCTs) | ⨁◯◯◯ Very low |
| Mortality | - | | | | Not reported |
| Quality of life | - | | | | Not reported |
| Cost | - | | | | Not reported |
| Adverse events | - | | | | Not reported |

| **S13 Table: Mistiaen and Poot, 2006: TFU compared to usual care for surgery patients** | | | | | |
| --- | --- | --- | --- | --- | --- |
| **Patient or population:** surgery patients  **Setting:** hospital to home  **Intervention:** TFU  **Comparison:** usual care | | | | | |
| Outcomes | **Anticipated absolute effects^*^** (95% CI) | | Relative effect (95% CI) | № of participants (studies) | Certainty of the evidence (GRADE) |
|  | **Risk with usual care** | **Risk with TFU** |  |  |  |
| Readmission | - | - | **RR 0.65** (0.28 to 1.55) | (5 RCTs) | ⨁◯◯◯ Very low |
| ED visit | - | - | **RR 1.47** (0.85 to 2.53) | (4 RCTs) | ⨁◯◯◯ Very low |
| Satisfaction | Five studies found no differences in satisfaction and only Fallis 2001 concludes that the TFU group is statistically more satisfied. | |  | (6 RCTs) | ⨁◯◯◯ Very low |
| Mortality | - | | | | Not reported |
| Quality of life | - | | | | Not reported |
| Cost | - | | | | Not reported |
| Adverse events | - | | | | Not reported |

| **S14 Table: Mistiaen and Poot, 2006: TFU compared to usual care for cardiac patients** | | | | | |
| --- | --- | --- | --- | --- | --- |
| **Patient or population:** cardiac patients  **Setting:** hospital to home  **Intervention:** TFU  **Comparison:** usual care | | | | | |
| Outcomes | **Anticipated absolute effects^*^** (95% CI) | | Relative effect (95% CI) | № of participants (studies) | Certainty of the evidence (GRADE) |
|  | **Risk with usual care** | **Risk with TFU** |  |  |  |
| Readmission | - | | **RR 0.75** (0.41 to 1.36) | (4 RCTs) | ⨁◯◯◯ Very low |
| Satisfaction | There were no statistical differences. | |  | (5 RCTs) | ⨁◯◯◯ Very low |
| Compliance | There were no statistical differences. | |  | (2 RCTs) | ⨁◯◯◯ Very low |
| Mortality | - | | | | Not reported |
| Quality of life | - | | | | Not reported |
| Cost | - | | | | Not reported |
| Adverse events | - | | | | Not reported |

| **S15 Table: Mistiaen and Poot, 2006: TFU compared to usual care for cardiac surgery patients** | | | | | |
| --- | --- | --- | --- | --- | --- |
| **Patient or population:** cardiac surgery patients  **Setting:** hospital to home  **Intervention:** TFU  **Comparison:** usual care | | | | | |
| Outcomes | **Anticipated absolute effects^*^** (95% CI) | | Relative effect (95% CI) | № of participants (studies) | Certainty of the evidence (GRADE) |
|  | **Risk with usual care** | **Risk with TFU** |  |  |  |
| Readmission | - | | | | Not reported |
| Anxiety | - | | **MD -0.47** (-1.28 to 0.34) | (3 RCTs) | ⨁◯◯◯ Very low |
| Depression | They were two studies, both studies found no statistically significant difference between intervention and control groups | |  | (2 RCTs) | ⨁◯◯◯ Very low |
| Compliance | - | | **RR 1.86** (0.59 to 4.78) | (2 RCTs) | ⨁◯◯◯ Very low |
| Mortality | - | | | | Not reported |
| Quality of life | - | | | | Not reported |
| Cost | - | | | | Not reported |
| Adverse events | - | | | | Not reported |

| **S16 Table: Holzingera F et al., 2017: discharge management strategies and post-discharge care interventions compared to usual care for the depressed patient** | | | | | |
| --- | --- | --- | --- | --- | --- |
| **Patient or population:** depressed patient  **Setting:** hospital to home  **Intervention:** discharge management strategies and post-discharge care interventions  **Comparison:** usual care | | | | | |
| Outcomes | **Anticipated absolute effects*** (95% CI) | | Relative effect (95% CI) | № of participants (studies) | Certainty of the evidence (GRADE) |
|  | **Risk with usual care** | **Risk with discharge management strategies and post-discharge care interventions** |  |  |  |
| Readmissions | 402 per 1,000 | 262 per 1,000 (169 to 406) | **RR 0.65** (0.42 to 1.01) | 710 (8 RCTs) | ⨁◯◯◯ Very low |
| Depression symptoms | 415 per 1,000 | -37 per 1,000 (-153 to 79) | **Std. Mean Difference** -0.09 (-0.37 to 0.19) | 592 (7 RCTs) | ⨁◯◯◯ Very low |
| Mortality | There is no significant for group difference | |  | (5 RCTs) | ⨁◯◯◯ Very low |
| Quality of life | There is no significant for group difference. | |  | (2 RCTs) | ⨁◯◯◯ Very low |
| Cost | - | | | | Not reported |
| Adverse events | - | | | | Not reported |

**RCT = randomized control trial, TCIs = Transitional care interventions, ED = Emergency Department, HF = Heart Failure, TFU = Telephone follow-up, CI= Confident Interval, RR= Relative Ratio, MLHFQ: Minnesota Living with Heart Failure Questionnaire, QALY: quality-adjusted life year; QoL: quality of life**

**GRADE Working Group grades of evidence**

**High quality**: we are very confident that the true effect lies close to that of the estimate of the effect.

**Moderate quality**: we are moderately confident in the effect estimate: the true effect is likely to be close to the estimate of the effect, but there is a possibility that it is substantially different.

**Low quality**: our confidence in the effect estimate iss limited: the true effect may be substantially different from the estimate of the effect.

**Very lows quality**: we have very little confidence in the effect estimate: the true effect is likely to be substantially different from the estimate of the effect
